# Synaptic Vesicle Proteins and Active Zone Plasticity

**DOI:** 10.3389/fnsyn.2016.00008

**Published:** 2016-04-18

**Authors:** Robert J. Kittel, Manfred Heckmann

**Affiliations:** Department of Neurophysiology, Institute of Physiology, Julius-Maximilians-University WürzburgWürzburg, Germany

**Keywords:** synaptotagmin I, Rab3, cytomatrix at the active zone, synaptic transmission and plasticity, synaptic vesicle, active zone, neurotransmitter release

## Abstract

Neurotransmitter is released from synaptic vesicles at the highly specialized presynaptic active zone (AZ). The complex molecular architecture of AZs mediates the speed, precision and plasticity of synaptic transmission. Importantly, structural and functional properties of AZs vary significantly, even for a given connection. Thus, there appear to be distinct AZ states, which fundamentally influence neuronal communication by controlling the positioning and release of synaptic vesicles. Vice versa, recent evidence has revealed that synaptic vesicle components also modulate organizational states of the AZ. The protein-rich cytomatrix at the active zone (CAZ) provides a structural platform for molecular interactions guiding vesicle exocytosis. Studies in *Drosophila* have now demonstrated that the vesicle proteins Synaptotagmin-1 (Syt1) and Rab3 also regulate glutamate release by shaping differentiation of the CAZ ultrastructure. We review these unexpected findings and discuss mechanistic interpretations of the reciprocal relationship between synaptic vesicles and AZ states, which has heretofore received little attention.

## Introduction

Chemical synapses are important regulators of neuronal information transfer. At these specialized intercellular contact sites the arrival of an action potential at the presynaptic terminal triggers the release of neurotransmitter onto a postsynaptic cell, where subsequent receptor activation gives rise to signal transduction. Remarkable electrophysiological work by Bernard Katz and colleagues on the quantal nature of neurotransmitter release in the mid-20th century set the basis for interpreting morphological features of the synaptic ultrastructure (Fatt and Katz, 1952; del Castillo and Katz, 1954; Palay, 1956; Couteaux and Pécot-Dechavassine, 1970). This combination of functional and structural studies helped to establish that transmitter is packaged into synaptic vesicles and discharged at a morphological specialization of the presynapse termed the active zone (AZ).

AZs transform a presynaptic action potential into the release of a chemical signal with high spatial and temporal precision. To perform this task, different proteins, some ubiquitously expressed and some highly specialized, are recruited to the AZ. Here, these molecules act in concert to control the final stages of the synaptic vesicle cycle. Vesicles are guided to the AZ membrane, docked and primed in a release-ready state and fused with the plasma membrane upon calcium ion (Ca^2+^) influx through voltage-gated Ca^2+^ channels (VGCCs). To ensure spatial precision of exocytosis, molecular interactions spanning the synaptic cleft align the AZ membrane exactly opposite the postsynaptic receptor field. The impressive speed and precise timing of neurotransmitter release, in turn, is provided by the coordinated interplay of individual protein-protein interactions occurring at the AZ (Jahn and Fasshauer, 2012). In addition to the core fusion complex, containing SNARE (“soluble NSF-attachment protein receptor”) and SM (“Sec1/Munc18-like”) proteins, vesicle components and AZ-specific proteins contribute to these interactions and help to position synaptic vesicles in close proximity to Ca^2+^ channels. The operation of these molecular machines enables presynaptic Ca^2+^ inflow to be followed by a postsynaptic current in less than a millisecond (Geiger and Jonas, 2000).

Current evidence suggests that the central protein complex surrounding VGCCs at *Drosophila* AZs is made up of RIM (Graf et al., 2012; Müller et al., 2012), RIM-BP (RIM-binding protein; Liu et al., 2011), Unc13 (Aravamudan et al., 1999), Liprin-α (Kaufmann et al., 2002; Fouquet et al., 2009), Syd-1 (Owald et al., 2010), Fife (homolog of vertebrate Piccolo; Bruckner et al., 2012) and Brp (Bruchpilot; Kittel et al., 2006b; Wagh et al., 2006). The membrane-proximal N-terminal domain of Brp is homologous to the vertebrate AZ component CAST/ELKS/ERC (CAST hereafter), while its coiled-coil rich C-terminus, which reaches into the cell interior, is related to large cytoskeletal proteins and is not conserved in its shorter ortholog (Wagh et al., 2006; Fouquet et al., 2009). The large vertebrate AZ scaffolding protein Bassoon does not appear to be encoded by the *Drosophila* genome. This has led to the suggestion that by tethering synaptic vesicles to the cytomatrix at the active zone (CAZ; via its C-term) and by clustering AZ VGCCs (via its N-term) Brp incorporates the functions of several vertebrate AZ proteins thereby ensuring efficient excitation-secretion coupling (Kittel et al., 2006a; Hallermann et al., 2010c).

In electron micrographs, the protein complexes assembled at the AZ are depicted as electron-dense material on the presynaptic plasma membrane. This CAZ often includes prominent structures reaching into the cytoplasm, which vary considerably between different synapses in a species- and cell-type specific manner (Zhai and Bellen, 2004). While chemical synapses operate by the same basic principle (Katz, 1970) the ultrastructural diversity emphasizes the non-uniform protein composition and organization of AZs. This observation raises the question how these complex molecular architectures are mechanistically linked to diverse functional adaptations of synaptic neurotransmitter release (Atwood and Karunanithi, 2002).

Here we follow the hypothesis that discrete organizational states can be specified for AZs. At present it is unclear how many such states may exist, what their functional significance is, or whether AZ differences may turn out to be more appropriately described by a continuum. However, for the time being this heuristic mode of inquiry is a useful means to clarify organizational principles underlying an information processing system. The precise spatial arrangement of AZ proteins, i.e., their orientation relative to the membrane and other AZ molecules, their copy number and the stoichiometry of macromolecular complexes, is functionally relevant. We therefore suggest that studying the nanoscopic arrangement of core CAZ components, such as the large filamentous Brp protein, relative to other proteins like VGCCs can help to distinguish and interpret AZ physiology.

## Plasticity of AZ States

Structural features and functional properties of AZs differ between various neuron types, between individual synapses belonging to the same neuron and at one and the same site over time (Atwood and Karunanithi, 2002). This plasticity of AZ states is both a developmental phenomenon and can also occur in the mature nervous system in response to changes in synaptic activity. Generally speaking, synaptic plasticity can be divided into short-lived and long-term forms.

Functional changes at the AZ feature prominently in short-term synaptic plasticity (Hallermann et al., 2010b; Regehr, 2012). Synaptic transmission can undergo rapid facilitation during ongoing activity through the accumulation of free Ca^2+^ in the presynaptic terminal, thereby raising vesicle release probability (Katz and Miledi, 1968; Schneggenburger and Neher, 2005). Conversely, depression of transmitter release can occur on a short time scale due to an inactivation of VGCCs or a depletion of readily-releasable vesicles (RRVs; Forsythe et al., 1998; Neher, 2015). Thus, in addition to spatio-temporal Ca^2+^ dynamics, kinetics of vesicle recruitment, priming and AZ release site (re)generation play an important role in shaping short-term plasticity (Junge et al., 2004; Hallermann et al., 2010c; Neher, 2010).

The CAZ is a dynamic structure and its molecular reorganization can shape synaptic function on a time scale of minutes (Matz et al., 2010). Such AZ plasticity can be induced by artificial changes of synaptic activity (Wojtowicz et al., 1994; Spangler et al., 2013) and by natural stimuli. Particularly striking examples of CAZ remodeling *in vivo* have been observed in visual systems of flies and vertebrates where light-dark changes affect the CAZ ultrastructure of photoreceptors (Abe and Yamamoto, 1984; Rybak and Meinertzhagen, 1997; Spiwoks-Becker et al., 2004). Recent work in *Drosophila* has shown that light exposure triggers the removal of Brp, RIM-BP and Liprin-α from photoreceptor terminals, whereas VGCCs and Syd-1 remain unaffected by this molecular plasticity (Sugie et al., 2015).

Homeostatic synaptic plasticity describes a particular form of activity-dependent plasticity, which serves to maintain constant transmission strength in response to altered pre- or postsynaptic function (Davis and Müller, 2015). In an evolutionarily conserved homeostatic process, observed for example at end-plates of myasthenia gravis patients, reduced postsynaptic sensitivity is counteracted by upregulated neurotransmitter release to restore action potential-evoked postsynaptic current amplitudes and maintain muscle excitation (Cull-Candy et al., 1980; Wang et al., 2016). At the *Drosophila* neuromuscular junction (NMJ), molecular mechanisms underlying related modifications of AZ states have been studied in considerable detail. Here, a homeostatic enhancement of Ca^2+^ influx through VGCCs is mediated by RIM-BP and plasma membrane insertion of epithelial sodium channels (ENaC; Younger et al., 2013; Müller et al., 2015). The concurrent recruitment of RRVs is guided by RIM as well as RIM-BP and is accompanied by the enlargement of the Brp-positive CAZ (Weyhersmüller et al., 2011; Müller et al., 2015).

Activity-dependent, long-term synaptic plasticity plays an important role in complex brain functions and represents a likely cellular correlate of memory formation. Mossy fiber synapses in the mammalian hippocampus undergo presynaptically expressed long-term potentiation (LTP), which requires the CAZ constituent RIM1α and the vesicle protein Rab3A (Castillo et al., 1997, 2002). Interactions between RIM1α with Rab3A and VGCCs promote tight Ca^2+^ channel-vesicle coupling (Han et al., 2011; Kaeser et al., 2011) and this process has been suggested to underlie increased transmitter release during mossy fiber LTP (Nicoll and Schmitz, 2005). However, details and direct evidence to support this hypothesis have not yet been presented. In *Drosophila*, odor memory formation is associated with presynaptic plasticity of Kenyon cells, the intrinsic mushroom body neurons (Heisenberg, 2003). Moreover, recent work has uncovered long-term synaptic depression in the context of aversive olfactory learning, consistent with functional modifications of Kenyon cell AZs (Hige et al., 2015). Brp and the vesicle-associated phosphoprotein Synapsin have been implicated in associative learning (Godenschwege et al., 2004; Knapek et al., 2011), though here too, we still lack basic information on the molecular mechanisms underlying memory-related changes of AZ states.

Developmental processes can also target the molecular architecture of AZs giving rise to changes in synaptic strength. For example, at several synapses of the mammalian auditory pathway, developmental changes affect the coupling distance between RRVs and VGCCs at the AZ (Fedchyshyn and Wang, 2005; Wong et al., 2014). Whereas immature AZs display loose coupling, maturation tightens the spatial association of VGCCs with membrane-docked vesicles. By placing the vesicular Ca^2+^ sensor closer to the source of Ca^2+^ influx, this conversion from “microdomain” to “nanodomain” coupling regimes promotes transmission efficiency by increasing neurotransmitter release probability (Eggermann et al., 2011).

As individual synapses mature during the development of the glutamatergic *Drosophila* NMJ, the molecular complexity and Brp content of their AZs increase (Schmid et al., 2008; Fouquet et al., 2009). Brp helps cluster presynaptic VGCCs and, correspondingly, an AZ’s neurotransmitter release probability correlates with Brp protein copy number (Kittel et al., 2006b; Ehmann et al., 2014). The developmental incorporation of Brp therefore likely promotes synaptic strength. Interestingly, Brp recruitment is accompanied by changes to the glutamate receptor subunit composition of an AZ’s postsynaptic partner. As the AZ grows, GluR-IIA accumulation is reduced and receptor incorporation shifts towards GluR-IIB (Schmid et al., 2008). This transsynaptic relationship appears to be bidirectional, since* brp* mutants possess elevated GluR-IIA levels and *gluR-IIA* mutants display increased release and elevated Brp levels (DiAntonio et al., 1999; Weyhersmüller et al., 2011). Hence, heterogeneous AZ states are matched with the molecular makeup of postsynaptic receptor fields.

The differentiation of this synaptic system operates at multiple levels of organization. Certain AZs participate in spontaneous and evoked neurotransmitter release, while others preferentially support one mode of exocytosis or the other, possibly depending on their maturation state (Melom et al., 2013; Peled et al., 2014). Moreover, a structural and functional gradient develops along a specific larval motoneuron. Distal boutons of the “type Ib” neuron are larger than their proximal counterparts, they possess more AZs and these, in turn, contain more Brp molecules (Ehmann et al., 2014; Paul et al., 2015). Accordingly, action potentials in distal boutons generate larger Ca^2+^ signals and release a greater number of synaptic vesicles in a more synchronized manner (Guerrero et al., 2005; Peled and Isacoff, 2011; Paul et al., 2015).

## Molecular Manipulations of Active Zone States

### CAZ Proteins

The function of individual CAZ proteins has been deduced mainly by studying mutant alleles. In addition to functional phenotypes, such molecular manipulations may also modify the AZ ultrastructure. These changes are most readily detected at synaptic contacts with prominent CAZ architectures visible in electron micrographs, such as ribbons of vertebrate sensory synapses or the T-bar at the *Drosophila* NMJ.

Brp is an integral component of the *Drosophila* T-bar and is essential for its assembly. At *brp* null mutant AZs VGCCs are mislocalized and T-bars are missing (Kittel et al., 2006b). T-bars appear truncated in C-terminal deletion mutants (Fouquet et al., 2009) and are misshaped when posttranslational modification of Brp is disturbed (Miśkiewicz et al., 2011). In the absence of RIM-BP, which acts in concert with Brp to cluster VGGCs and shape the T-bar, Brp-positive CAZ structures are severely misformed (Liu et al., 2011). Besides these core determinants of T-bar morphology, other CAZ proteins also define the T-bar ultrastructure, reflecting the intricate protein complex of the CAZ. Whereas T-bars appear “overgrown” in mutants of *liprin-α* and *syd-1* (Kaufmann et al., 2002; Owald et al., 2010), *fife* mutant AZs display membrane disruptions and detached T-bar-like structures (Bruckner et al., 2012).

Turning to vertebrate sensory synapses, genetic studies in mouse have uncovered contributions of several CAZ components to ribbon morphology. While Bassoon, a large protein component of the CAZ, is involved in attaching ribbons to the AZ membrane in photoreceptors (Dick et al., 2003) and cochlear inner hair cells (Khimich et al., 2005), Piccolino, a ribbon-specific splice variant of the CAZ protein Piccolo, appears to shape the ribbon ultrastructure (Regus-Leidig et al., 2014). CAST in turn exerts a more subtle influence on the CAZ architecture. In rod photoreceptors of CAST knock-out mice ribbons are shorter despite an apparently intact overall molecular organization (tom Dieck et al., 2012).

Disrupting expression or proper function of a CAZ protein may change the spatial arrangement and operation of other AZ constituents. Analyzing this molecular organization, ideally quantitatively and in combination with physiological and biochemical data, can provide insight to complex protein interactions at the CAZ. With rearrangements taking place on the nanometer scale, until quite recently, such changes to the molecular architecture have been difficult to detect. The introduction of super-resolution light microscopy to the Neurosciences is beginning to change this situation (Sigrist and Sabatini, 2012; Ehmann et al., 2015).

Since small changes to the physical distance between VGCCs and RRVs have a profound effect on neurotransmitter release (Eggermann et al., 2011) information on the nanoscopic distribution of VGCCs in the AZ membrane is important. In an interesting parallel, super-resolution microscopy has uncovered disarranged VGCC clusters at *brp* and *bassoon* mutant AZs of *Drosophila* and mouse, respectively (Kittel et al., 2006b; Frank et al., 2010; Hallermann et al., 2010c). Combining such modern light microscopy techniques with sophisticated electron microscopy (Indriati et al., 2013) opens new prospects of clarifying structure-function relationships of AZ states by resolving VGCC topographies with respect to synaptic vesicles and the CAZ.

### Synaptic Vesicle Proteins

The molecular architecture of the AZ is not only altered by interfering with CAZ components, but can also change when VGCCs (Urbano et al., 2003) or synaptic vesicle proteins are manipulated.

**Figure 1:**
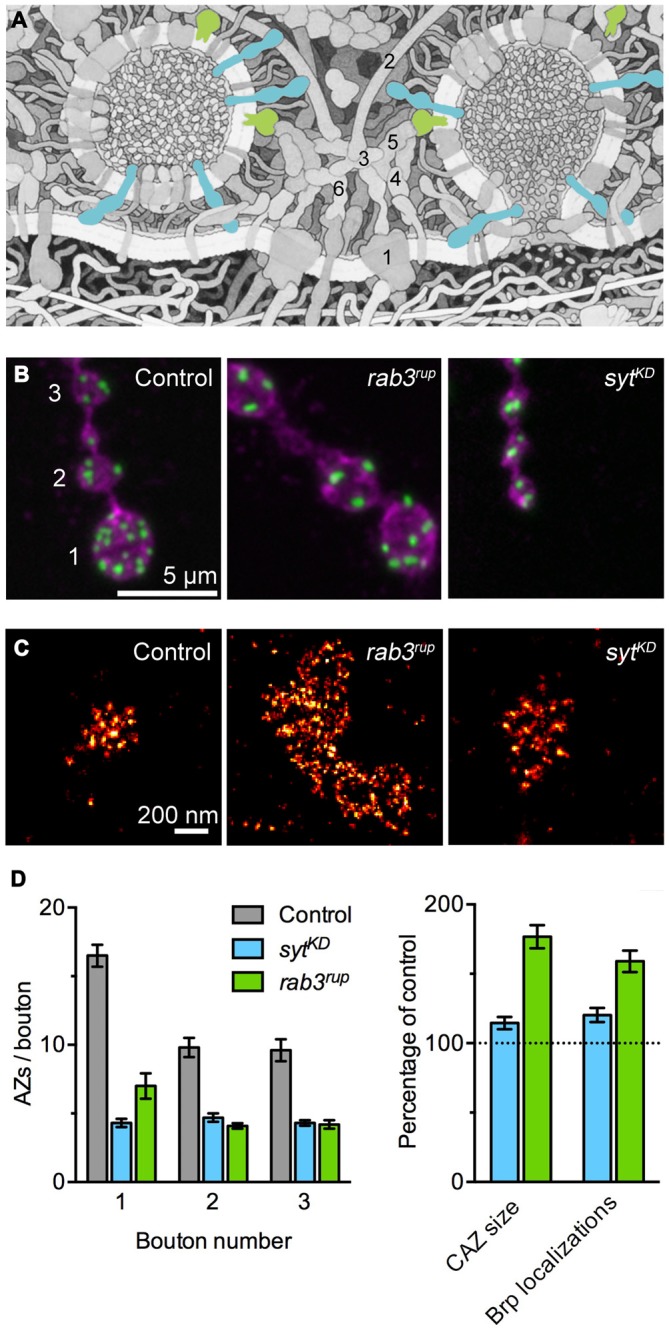
**Synaptotagmin-1 (Syt1) and Rab3 shape active zone (AZ) differentiation and ultrastructure. (A)** Illustration of the molecular complexity of the AZ. Syt1 (blue) and Rab3 (green) are highlighted. The numbers indicate core AZ proteins mentioned in the text: (1) VGCC; (2) Bassoon; (3) CAST; (4) Munc-13/18; (5) RIM; (6) Liprin-α. Modified from Goodsell (2009; © by The International Union of Biochemistry and Molecular Biology). **(B)** Shown are confocal images of the terminal three boutons along a type Ib axon branch. Staining against the membrane marker HRP (magenta) and Brp (green) illustrates the reduced number of AZs in *rab3* mutant (*rab3^rup^*) and *syt^KD^* motoneurons. Note the small boutons at the *syt^KD^* neuromuscular junction (NMJ). Taken from Paul et al. (2015). **(C)** Super-resolution imaging of Brp by *d*STORM (*direct* stochastic optical reconstruction microscopy; Heilemann et al., 2008). Examples of Brp organization at control and *syt^KD^* AZs (Paul et al., 2015) and the massively enlarged cytomatrix at the active zone (CAZ) frequently observed at *rab3^rup^* NMJs (Graf et al., 2009; Ehmann et al., 2014). **(D)** Quantification of the AZ gradient along type Ib motoneurons (left, related to **B**) and the nanoscopic organization of Brp at the CAZ (right, related to **C**). Summary of data presented in Ehmann et al. (2014) and Paul et al. (2015).

Rab3 is a small synaptic vesicle-associated GTPase involved in vesicle cycling, docking and exocytosis (Figure 1A; Südhof, 2004). In a seminal study on Rab3 function in *Drosophila*, Graf et al. reported that Rab3 also controls the protein composition of AZs. At *rab3* mutant NMJs the number of Brp positive AZs drops down to about 30%. At the same time, individual AZs containing Brp are dramatically enlarged, these sites frequently display multiple T-bars and accumulate VGCCs (Figures 1B–D; Graf et al., 2009). According to quantitative super-resolution imaging, the number of Brp molecules at the fraction of available sites is increased on average 1.6-fold and correspondingly release probability at these AZs increases with increasing Brp content (Peled and Isacoff, 2011; Ehmann et al., 2014). Interestingly, late expression of *rab3* can rescue the already manifested mutant phenotype, illustrating the dynamic control of Rab3 on the distribution and nucleation of Brp at AZs (Graf et al., 2009).

The vesicular protein Synaptotagmin-1 (Syt1) plays a decisive role as a Ca^2+^ sensor by triggering neurotransmitter secretion on the one hand and clamping vesicle fusion on the other (Figure 1A; Brose et al., 1992; DeBello et al., 1993). Recent work at the *Drosophila* NMJ has described a surprising additional influence of Syt1 on structural synaptic differentiation (Paul et al., 2015). Reducing Syt1 protein levels leads to major changes in the morphology of the type Ib motoneuron. Both bouton area and the number of AZs per bouton drop below 50% of wild type values, the Brp count per CAZ increases moderately, and the structure-function gradient is lost (Figures 1B–D). Proximal and distal boutons at *syt1* knock-down (*syt^KD^*) NMJs have uniform dimensions and they posses comparable numbers of AZs with similar Brp content. In agreement with these observations, focal electrophysiological measurements report indistinguishable evoked excitatory postsynaptic current amplitudes at proximal and distal locations.

It is recognized that CAZ components can affect the organization of synaptic vesicles at the AZ. For example, filamentous proteins concentrate vesicles in the vicinity of the AZ membrane by tethering them to the CAZ. Such a function is performed by the C-terminal end of Brp to promote rapid vesicle recruitment during high frequency synaptic activity (Hallermann et al., 2010c) and a related role has been ascribed to Bassoon (Hallermann et al., 2010a). It has also been reported that Syt2 participates in positioning vesicles close to VGCCs at the AZ membrane (Young and Neher, 2009). However, to date little attention has been paid to the possibility that vesicle proteins such as Synaptotagmins may influence the structural organization of the AZ (Neher and Penner, 1994).

Work on Syt1 has focused on its role in regulating the final stages of the synaptic vesicle cycle and specifically on its Ca^2+^ -dependent control of exocytosis. In contrast, an additional influence of Syt1 on neuronal differentiation and AZ architecture has remained largely unaddressed. With increasing knowledge of the molecular makeup of the CAZ (Figure 1A) and information on its capacity to undergo dynamic rearrangements, further lines of investigation can now be followed up (Graf et al., 2009; Paul et al., 2015). To this end, modern light microscopy methods provide new options for interrogating the involvement of synaptic vesicle proteins in (ultra)structural organization (Figure 1C).

## Outlook

There is strong evidence that the CAZ is a plastic structure and that its dynamic rearrangement gives rise to different functional properties of AZs. The observations that synaptic vesicle proteins can influence this plasticity are puzzling (Graf et al., 2009; Paul et al., 2015) and suggest a connection between vesicle dynamics and CAZ assembly (Chen et al., 2015). The mechanisms through which Syt1 and Rab3 shape the CAZ are currently not understood. In principle, alterations of CAZ structure and synaptic differentiation may reflect compensatory, homeostatic changes triggered by impaired presynaptic function. However, the structural phenotypes of *syt^KD^* and* rab3* mutant NMJs are dissimilar, indicating different pathways, and other manipulations of vesicle release do not parallel the Brp layout seen e.g., in *rab3* mutants (Graf et al., 2009).

Alternatively, the structural abnormalities may be directly linked to the involvement of Syt1 and Rab3 in the synaptic vesicle cycle. Consistent with this notion, a recent study of mutant alleles has demonstrated that normal AZ differentiation depends on a typical vesicle tethering mechanism of Rab3 (Chen et al., 2015). It will be of interest to carry out an analogous mutational analysis of *syt1* to identify protein domains relevant for shaping CAZ ultrastructure and neuronal morphology. Notably, Rab3 is enriched at AZs of the *Drosophila* NMJ. Its punctate clustering requires the presence of Brp and is quite different to the more homogeneous distribution of other synaptic vesicle proteins (Graf et al., 2009). This suggests that Rab3 is associated with a sub-population of vesicles in the vicinity of the CAZ, or with the CAZ itself. Future work will have to clarify how the specific localization pattern of Rab3 is mechanistically connected to its influence on AZ plasticity. An intriguing possibility is that Rab3 and Syt1 exert their structural effects through association with vesicle populations other than neurotransmitter-filled synaptic vesicles. Work in rodent neurons has suggested that preassembled CAZ complexes are transported to developing synapses in so-called Piccolo-Bassoon transport vesicles (PTVs), which include e.g., Piccolo, Bassoon, Munc-13, RIM, CAST and VGCCs (Zhai et al., 2001; Shapira et al., 2003; Maas et al., 2012). Whereas Rab3a is associated with PTVs (Shapira et al., 2003), Syt1 instead appears to be included in synaptic vesicle protein transport vesicles (STVs), which are transported together with PTVs in a coordinated manner (Zhai et al., 2001; Tao-Cheng, 2007; Bury and Sabo, 2011). In *Drosophila*, Brp is co-transported along the axon with RIM-BP (Siebert et al., 2015). However, neither Liprin-α and Syd-1, which precede Brp during AZ assembly (Fouquet et al., 2009), nor Rab3 are associated with this putative precursor complex.

Why have genetic studies of *rab3* and *syt1* in other organisms not reported structural AZ defects similar to those observed in *Drosophila*? One possibility is that the stereotypic morphological layout of the fly NMJ, including its developmental synaptic differentiation, facilitates the quantification of parameters pertaining to neuronal structure and AZ ultrastructure, which are more difficult to measure in other systems. In particular, the T-bar as a prominent marker of the CAZ and the characteristic modular assembly of Brp support analyses of the AZ nanostructure (Kittel et al., 2006b; Graf et al., 2009; Liu et al., 2011; Ehmann et al., 2014; Paul et al., 2015). To clarify whether the structural roles of synaptic vesicle proteins are a peculiarity of the *Drosophila* NMJ or an evolutionarily conserved feature, it will be worthwhile to investigate other synapses with prominent CAZ architectures.

Finally, these new results have important implications for our current understanding of Rab3 and Syt1 functions. Single-synapse resolution is rarely attained in electrophysiological recordings and therefore structural data, e.g., concerning the number of sampled synapses, must be taken into account when extending functional interpretations to the single synapse level. Evidently, we still lack fundamental information on the mechanisms guiding the dynamic organization of AZ states. As we continue filling the gaps old players may be seen in a new light.

## Author Contributions

RJK and MH conceived and wrote the manuscript.

## Conflict of Interest Statement

The authors declare that the research was conducted in the absence of any commercial or financial relationships that could be construed as a potential conflict of interest.

## References

[B1] Abe H. Yamamoto T. Y. (1984). Diurnal changes in synaptic ribbons of rod cells of the turtle. J. Ultrastruct. Res. 86, 246–251. 10.1016/s0022-5320(84)90104-7 6544862

[B2] Aravamudan B. Fergestad T. Davis W. S. Rodesch C. K. Broadie K. (1999). *Drosophila* UNC-13 is essential for synaptic transmission. Nat. Neurosci. 2, 965–971. 10526334 10.1038/14764

[B3] Atwood H. L. Karunanithi S. (2002). Diversification of synaptic strength: presynaptic elements. Nat. Rev. Neurosci. 3, 497–516. 10.1038/nrn876 12094207

[B4] Brose N. Petrenko A. G. Südhof T. C. Jahn R. (1992). Synaptotagmin: a calcium sensor on the synaptic vesicle surface. Science 256, 1021–1025. 10.1126/science.1589771 1589771

[B5] Bruckner J. J. Gratz S. J. Slind J. K. Geske R. R. Cummings A. M. Galindo S. E.. (2012). Fife, a *Drosophila* Piccolo-RIM homolog, promotes active zone organization and neurotransmitter release. J. Neurosci. 32, 17048–17058. 10.1523/JNEUROSCI.3267-12.2012 23197698 PMC3524967

[B6] Bury L. A. D. Sabo S. L. (2011). Coordinated trafficking of synaptic vesicle and active zone proteins prior to synapse formation. Neural. Dev. 6:24. 10.1186/1749-8104-6-24 21569270 PMC3103415

[B7] Castillo P. E. Janz R. Südhof T. C. Tzounopoulos T. Malenka R. C. Nicoll R. A. (1997). Rab3A is essential for mossy fibre long-term potentiation in the hippocampus. Nature 388, 590–593. 10.1038/41574 9252190

[B8] Castillo P. Schoch S. Schmitz F. Südhof T. Malenka R. (2002). RIM1α is required for presynaptic long-term potentiation. Nature 415, 327–330. 10.1038/415327a 11797010

[B9] Chen S. Gendelman H. K. Roche J. P. Alsharif P. Graf E. R. (2015). Mutational analysis of Rab3 function for controlling active zone protein composition at the *Drosophila* neuromuscular junction. PLoS One 10:e0136938. 10.1371/journal.pone.0136938 26317909 PMC4552854

[B10] Couteaux R. Pécot-Dechavassine M. (1970). Synaptic vesicles and pouches at the level of “active zones” of the neuromuscular junction. C. R. Hebd. Seances Acad. Sci. Ser. D Sci. Nat. 271, 2346–2349. 4995202

[B11] Cull-Candy S. G. Miledi R. Trautmann A. Uchitel O. D. (1980). On the release of transmitter at normal, myasthenia gravis and myasthenic syndrome affected human end-plates. J. Physiol. 299, 621–638. 10.1113/jphysiol.1980.sp013145 6103954 PMC1279245

[B12] Davis G. W. Müller M. (2015). Homeostatic control of presynaptic neurotransmitter release. Annu. Rev. Physiol. 77, 251–270. 10.1146/annurev-physiol-021014-071740 25386989

[B13] DeBello W. M. Betz H. Augustine G. J. (1993). Synaptotagmin and neurotransmitter release. Cell 74, 947–950. 10.1016/0092-8674(93)90716-4 8104706

[B14] del Castillo J. Katz B. (1954). Quantal components of the end-plate potential. J. Physiol. 124, 560–573. 10.1113/jphysiol.1954.sp005129 13175199 PMC1366292

[B15] DiAntonio A. Petersen S. A. Heckmann M. Goodman C. S. (1999). Glutamate receptor expression regulates quantal size and quantal content at the *Drosophila* neuromuscular junction. J. Neurosci. 19, 3023–3032. 10191319 10.1523/JNEUROSCI.19-08-03023.1999 PMC6782296

[B16] Dick O. tom Dieck S. Altrock W. D. Ammermüller J. Weiler R. Garner C. C.. (2003). The presynaptic active zone protein bassoon is essential for photoreceptor ribbon synapse formation in the retina. Neuron 37, 775–786. 10.1016/s0896-6273(03)00086-2 12628168

[B17] Eggermann E. Bucurenciu I. Goswami S. P. Jonas P. (2011). Nanodomain coupling between Ca^2+^ channels and sensors of exocytosis at fast mammalian synapses. Nat. Rev. Neurosci. 13, 7–21. 10.1038/nrn3125 22183436 PMC3617475

[B18] Ehmann N. Sauer M. Kittel R. J. (2015). Super-resolution microscopy of the synaptic active zone. Front. Cell. Neurosci. 9:7. 10.3389/fncel.2015.00007 25688186 PMC4311638

[B19] Ehmann N. van de Linde S. Alon A. Ljaschenko D. Keung X. Z. Holm T.. (2014). Quantitative super-resolution imaging of Bruchpilot distinguishes active zone states. Nat. Commun. 5, 4650–4661. 10.1038/ncomms5650 25130366 PMC4143948

[B20] Fatt P. Katz B. (1952). Spontaneous subthreshold activity at motor nerve endings. J. Physiol. 117, 109–128. 14946732 PMC1392564

[B21] Fedchyshyn M. J. Wang L.-Y. (2005). Developmental transformation of the release modality at the calyx of held synapse. J. Neurosci. 25, 4131–4140. 10.1523/jneurosci.0350-05.2005 15843616 PMC6724960

[B22] Forsythe I. D. Tsujimoto T. Barnes-Davies M. Cuttle M. F. Takahashi T. (1998). Inactivation of presynaptic calcium current contributes to synaptic depression at a fast central synapse. Neuron 20, 797–807. 10.1016/s0896-6273(00)81017-x 9581770

[B23] Fouquet W. Owald D. Wichmann C. Mertel S. Depner H. Dyba M.. (2009). Maturation of active zone assembly by *Drosophila* Bruchpilot. J. Cell Biol. 186, 129–145. 10.1083/jcb.200812150 19596851 PMC2712991

[B24] Frank T. Rutherford M. A. Strenzke N. Neef A. Pangršič T. Khimich D.. (2010). Bassoon and the synaptic ribbon organize Ca^2+^ channels and vesicles to add release sites and promote refilling. Neuron 68, 724–738. 10.1016/j.neuron.2010.10.027 21092861 PMC3005353

[B25] Geiger J. R. Jonas P. (2000). Dynamic control of presynaptic Ca^2+^ inflow by fast-inactivating K^+^ channels in hippocampal mossy fiber boutons. Neuron 28, 927–939. 10.1016/s0896-6273(00)00164-1 11163277

[B26] Godenschwege T. A. Reisch D. Diegelmann S. Eberle K. Funk N. Heisenberg M.. (2004). Flies lacking all synapsins are unexpectedly healthy but are impaired in complex behaviour. Eur. J. Neurosci. 20, 611–622. 10.1111/j.1460-9568.2004.03527.x 15255973

[B27] Goodsell D. S. (2009). Neuromuscular synapse. Biochem. Mol. Biol. Educ. 37, 204–210. 10.1002/bmb.20297 21567738

[B28] Graf E. R. Daniels R. W. Burgess R. W. Schwarz T. L. Diantonio A. (2009). Rab3 dynamically controls protein composition at active zones. Neuron 64, 663–677. 10.1016/j.neuron.2009.11.002 20005823 PMC2796257

[B29] Graf E. R. Valakh V. Wright C. M. Wu C. Liu Z. Zhang Y. Q.. (2012). RIM promotes calcium channel accumulation at active zones of the *Drosophila* neuromuscular junction. J. Neurosci. 32, 16586–16596. 10.1523/JNEUROSCI.0965-12.2012 23175814 PMC3516196

[B30] Guerrero G. Rieff D. F. Agarwal G. Ball R. W. Borst A. Goodman C. S.. (2005). Heterogeneity in synaptic transmission along a *Drosophila* larval motor axon. Nat. Neurosci. 8, 1188–1196. 10.1038/nn1526 16116446 PMC1402256

[B31] Hallermann S. Fejtova A. Schmidt H. Weyhersmüller A. Silver R. A. Gundelfinger E. D.. (2010a). Bassoon speeds vesicle reloading at a central excitatory synapse. Neuron 68, 710–723. 10.1016/j.neuron.2010.10.026 21092860 PMC3004039

[B32] Hallermann S. Heckmann M. Kittel R. J. (2010b). Mechanisms of short-term plasticity at neuromuscular active zones of *Drosophila*. HFSP J. 4, 72–84. 10.2976/1.3338710 20811513 PMC2931299

[B33] Hallermann S. Kittel R. J. Wichmann C. Weyhersmüller A. Fouquet W. Mertel S.. (2010c). Naked dense bodies provoke depression. J. Neurosci. 30, 14340–14345. 10.1523/JNEUROSCI.2495-10.2010 20980589 PMC6634796

[B34] Han Y. Kaeser P. S. Südhof T. C. Schneggenburger R. (2011). RIM determines Ca^2+^ channel density and vesicle docking at the presynaptic active zone. Neuron 69, 304–316. 10.1016/j.neuron.2010.12.014 21262468 PMC3259453

[B35] Heilemann M. van de Linde S. Schüttpelz M. Kasper R. Seefeldt B. Mukherjee A.. (2008). Subdiffraction-resolution fluorescence imaging with conventional fluorescent probes. Angew. Chem. Int. Ed. Engl. 47, 6172–6176. 10.1002/anie.200802376 18646237

[B36] Heisenberg M. (2003). Mushroom body memoir: from maps to models. Nat. Rev. Neurosci. 4, 266–275. 10.1038/nrn1074 12671643

[B37] Hige T. Aso Y. Modi M. N. Rubin G. M. Turner G. C. (2015). Heterosynaptic plasticity underlies aversive olfactory learning in *Drosophila*. Neuron 88, 985–998. 10.1016/j.neuron.2015.11.003 26637800 PMC4674068

[B38] Indriati D. W. Kamasawa N. Matsui K. Meredith A. L. Watanabe M. Shigemoto R. (2013). Quantitative localization of Cav2.1 (P/Q-type) voltage-dependent calcium channels in Purkinje cells: somatodendritic gradient and distinct somatic coclustering with calcium-activated potassium channels. J. Neurosci. 33, 3668–3678. 10.1523/JNEUROSCI.2921-allow12.2013 23426693 PMC4031662

[B39] Jahn R. Fasshauer D. (2012). Molecular machines governing exocytosis of synaptic vesicles. Nature 490, 201–207. 10.1038/nature11320 23060190 PMC4461657

[B40] Junge H. J. Rhee J.-S. Jahn O. Varoqueaux F. Spiess J. Waxham M. N.. (2004). Calmodulin and Munc13 form a Ca^2+^ sensor/effector complex that controls short-term synaptic plasticity. Cell 118, 389–401. 10.1016/j.cell.2004.06.029 15294163

[B41] Kaeser P. S. Deng L. Wang Y. Dulubova I. Liu X. Rizo J.. (2011). RIM proteins tether Ca^2+^ channels to presynaptic active zones via a direct PDZ-domain interaction. Cell 144, 282–295. 10.1016/j.cell.2010.12.029 21241895 PMC3063406

[B42] Katz B. (1970). “Nobel lecture: on the quantal mechanism of neural transmitter release,” in Nobel Lectures, Physiology or Medicine, 1963–1970, ed. Lindsten J. (Singapore: World Scientific Publishing Co.), 485–492.

[B43] Katz B. Miledi R. (1968). The role of calcium in neuromuscular facilitation. J. Physiol. 195, 481–492. 10.1113/jphysiol.1968.sp008469 4296699 PMC1351674

[B44] Kaufmann N. DeProto J. Ranjan R. Wan H. Van Vactor D. (2002). *Drosophila* liprin-alpha and the receptor phosphatase Dlar control synapse morphogenesis. Neuron 34, 27–38. 10.1016/s0896-6273(02)00643-8 11931739

[B45] Khimich D. Nouvian R. Pujol R. tom Dieck S. Egner A. Gundelfinger E. D.. (2005). Hair cell synaptic ribbons are essential for synchronous auditory signalling. Nature 434, 889–894. 10.1038/nature03418 15829963

[B46] Kittel R. J. Hallermann S. Thomsen S. Wichmann C. Sigrist S. J. Heckmann M. (2006a). Active zone assembly and synaptic release. Biochem. Soc. Trans. 34, 939–941. 10.1042/bst0340939 17052232

[B47] Kittel R. J. Wichmann C. Rasse T. M. Fouquet W. Schmidt M. Schmid A.. (2006b). Bruchpilot promotes active zone assembly, Ca^2+^ channel clustering and vesicle release. Science 312, 1051–1054. 10.1126/science.1126308 16614170

[B48] Knapek S. Sigrist S. Tanimoto H. (2011). Bruchpilot, a synaptic active zone protein for anesthesia-resistant memory. J. Neurosci. 31, 3453–3458. 10.1523/JNEUROSCI.2585-10.2011 21368057 PMC6623931

[B49] Liu K. S. Y. Siebert M. Mertel S. Knoche E. Wegener S. Wichmann C.. (2011). RIM-binding protein, a central part of the active zone, is essential for neurotransmitter release. Science 334, 1565–1569. 10.1126/science.1212991 22174254

[B50] Maas C. Torres V. I. Altrock W. D. Leal-Ortiz S. Wagh D. Terry-Lorenzo R. T.. (2012). Formation of Golgi-derived active zone precursor vesicles. J. Neurosci. 32, 11095–11108. 10.1523/JNEUROSCI.0195-12.2012 22875941 PMC3752076

[B51] Matz J. Gilyan A. Kolar A. McCarvill T. Krueger S. R. (2010). Rapid structural alterations of the active zone lead to sustained changes in neurotransmitter release. Proc. Natl. Acad. Sci. U S A 107, 8836–8841. 10.1073/pnas.0906087107 20421490 PMC2889309

[B52] Melom J. E. Akbergenova Y. Gavornik J. P. Littleton J. T. (2013). Spontaneous and evoked release are independently regulated at individual active zones. J. Neurosci. 33, 17253–17263. 10.1523/JNEUROSCI.3334-13.2013 24174659 PMC3812501

[B53] Miśkiewicz K. Jose L. E. Bento-Abreu A. Fislage M. Taes I. Kasprowicz J.. (2011). ELP3 controls active zone morphology by acetylating the ELKS family member Bruchpilot. Neuron 72, 776–788. 10.1016/j.neuron.2011.10.010 22153374

[B54] Müller M. Genç Ö. Davis G. W. (2015). RIM-binding protein links synaptic homeostasis to the stabilization and replenishment of high release probability vesicles. Neuron 85, 1056–1069. 10.1016/j.neuron.2015.01.024 25704950 PMC4354699

[B55] Müller M. Liu K. S. Y. Sigrist S. J. Davis G. W. (2012). RIM controls homeostatic plasticity through modulation of the readily-releasable vesicle pool. J. Neurosci. 32, 16574–16585. 10.1523/JNEUROSCI.0981-12.2012 23175813 PMC3523185

[B56] Neher E. (2010). What is rate-limiting during sustained synaptic activity: vesicle supply or the availability of release sites. Front. Syn. Neurosci. 2:144. 10.3389/fnsyn.2010.00144 21423530 PMC3059671

[B57] Neher E. (2015). Merits and limitations of vesicle pool models in view of heterogeneous populations of synaptic vesicles. Neuron 87, 1131–1142. 10.1016/j.neuron.2015.08.038 26402599

[B58] Neher E. Penner R. (1994). Mice sans synaptotagmin. Nature 372, 316–317. 10.1038/372316a0 7969483

[B59] Nicoll R. A. Schmitz D. (2005). Synaptic plasticity at hippocampal mossy fibre synapses. Nat. Rev. Neurosci. 6, 863–876. 10.1038/nrn1786 16261180

[B60] Owald D. Fouquet W. Schmidt M. Wichmann C. Mertel S. Depner H.. (2010). A Syd-1 homologue regulates pre- and postsynaptic maturation in *Drosophila*. J. Cell Biol. 188, 565–579. 10.1083/jcb.200908055 20176924 PMC2828917

[B61] Palay S. L. (1956). Synapses in the central nervous system. J. Biophys. Biochem. Cytol. 2, 193–202. 10.1083/jcb.2.4.193 13357542 PMC2229686

[B62] Paul M. M. Pauli M. Ehmann N. Hallermann S. Sauer M. Kittel R. J.. (2015). Bruchpilot and synaptotagmin collaborate to drive rapid glutamate release and active zone differentiation. Front. Cell. Neurosci. 9:29. 10.3389/fncel.2015.00029 25698934 PMC4318344

[B63] Peled E. S. Isacoff E. Y. (2011). Optical quantal analysis of synaptic transmission in wild-type and *rab3*-mutant *Drosophila* motor axons. Nat. Neurosci. 14, 519–526. 10.1038/nn.2767 21378971 PMC7645962

[B64] Peled E. S. Newman Z. L. Isacoff E. Y. (2014). Evoked and spontaneous transmission favored by distinct sets of synapses. Curr. Biol. 24, 484–493. 10.1016/j.cub.2014.01.022 24560571 PMC4017949

[B65] Regehr W. G. (2012). Short-term presynaptic plasticity. Cold Spring Harb. Perspect. Biol. 4:a005702 10.1101/cshperspect.a005702 22751149 PMC3385958

[B66] Regus-Leidig H. Fuchs M. Löhner M. Leist S. R. Leal-Ortiz S. Chiodo V. A.. (2014). *In vivo* knockdown of Piccolino disrupts presynaptic ribbon morphology in mouse photoreceptor synapses. Front. Cell. Neurosci. 8:259. 10.3389/fncel.2014.00259 25232303 PMC4153300

[B67] Rybak J. Meinertzhagen I. A. (1997). The effects of light reversals on photoreceptor synaptogenesis in the fly Musca domestica. Eur. J. Neurosci. 9, 319–333. 10.1111/j.1460-9568.1997.tb01402.x 9058052

[B68] Schmid A. Hallermann S. Kittel R. J. Khorramshahi O. Frölich A. M. J. Quentin C.. (2008). Activity-dependent site-specific changes of glutamate receptor composition *in vivo*. Nat. Neurosci. 11, 659–666. 10.1038/nn.2122 18469810

[B69] Schneggenburger R. Neher E. (2005). Presynaptic calcium and control of vesicle fusion. Curr. Opin. Neurobiol. 15, 266–274. 10.1016/j.conb.2005.05.006 15919191

[B70] Shapira M. Zhai R. G. Dresbach T. Bresler T. Torres V. I. Gundelfinger E. D.. (2003). Unitary assembly of presynaptic active zones from Piccolo-Bassoon transport vesicles. Neuron 38, 237–252. 10.1016/s0896-6273(03)00207-1 12718858

[B71] Siebert M. Böhme M. A. Driller J. H. Babikir H. Mampell M. M. Rey U.. (2015). A high affinity RIM-binding protein/Aplip1 interaction prevents the formation of ectopic axonal active zones. eLife 4:e06935. 10.7554/eLife.06935 26274777 PMC4536467

[B72] Sigrist S. J. Sabatini B. L. (2012). Optical super-resolution microscopy in neurobiology. Curr. Opin. Neurobiol. 22, 86–93. 10.1016/j.conb.2011.10.014 22051692

[B73] Spangler S. A. Schmitz S. K. Kevenaar J. T. de Graaff E. de Wit H. Demmers J.. (2013). Liprin-α2 promotes the presynaptic recruitment and turnover of RIM1/CASK to facilitate synaptic transmission. J. Cell Biol. 201, 915–928. 10.1083/jcb.201301011 23751498 PMC3678157

[B74] Spiwoks-Becker I. Glas M. Lasarzik I. Vollrath L. (2004). Mouse photoreceptor synaptic ribbons lose and regain material in response to illumination changes. Eur. J. Neurosci. 19, 1559–1571. 10.1111/j.1460-9568.2004.03198.x 15066152

[B75] Südhof T. C. (2004). The synaptic vesicle cycle. Annu. Rev. Neurosci. 27, 509–547. 10.1146/annurev.neuro.26.041002.131412 15217342

[B76] Sugie A. Hakeda-Suzuki S. Suzuki E. Silies M. Shimozono M. Möhl C.. (2015). Molecular remodeling of the presynaptic active zone of *Drosophila* photoreceptors via activity-dependent feedback. Neuron 86, 711–725. 10.1016/j.neuron.2015.03.046 25892303

[B77] Tao-Cheng J.-H. (2007). Ultrastructural localization of active zone and synaptic vesicle proteins in a preassembled multi-vesicle transport aggregate. Neuroscience 150, 575–584. 10.1016/j.neuroscience.2007.09.031 17977664 PMC2190624

[B78] tom Dieck S. Specht D. Strenzke N. Hida Y. Krishnamoorthy V. Schmidt K.-F.. (2012). Deletion of the presynaptic scaffold CAST reduces active zone size in rod photoreceptors and impairs visual processing. J. Neurosci. 32, 12192–12203. 10.1523/jneurosci.0752-12.2012 22933801 PMC6621541

[B79] Urbano F. J. Piedras-Rentería E. S. Jun K. Shin H.-S. Uchitel O. D. Tsien R. W. (2003). Altered properties of quantal neurotransmitter release at endplates of mice lacking P/Q-type Ca^2+^ channels. Proc. Natl. Acad. Sci. U S A 100, 3491–3496. 10.1073/pnas.0437991100 12624181 PMC152320

[B80] Wagh D. A. Rasse T. M. Asan E. Hofbauer A. Schwenkert I. Dürrbeck H.. (2006). Bruchpilot, a protein with homology to ELKS/CAST, is required for structural integrity and function of synaptic active zones in *Drosophila*. Neuron 49, 833–844. 10.1016/j.neuron.2006.02.008 16543132

[B81] Wang X. Pinter M. J. Rich M. M. (2016). Reversible recruitment of a homeostatic reserve pool of synaptic vesicles underlies rapid homeostatic plasticity of quantal content. J. Neurosci. 36, 828–836. 10.1523/JNEUROSCI.3786-15.2016 26791213 PMC4719018

[B82] Weyhersmüller A. Hallermann S. Wagner N. Eilers J. (2011). Rapid active zone remodeling during synaptic plasticity. J. Neurosci. 31, 6041–6052. 10.1523/JNEUROSCI.6698-10.2011 21508229 PMC6632979

[B83] Wojtowicz J. M. Marin L. Atwood H. L. (1994). Activity-induced changes in synaptic release sites at the crayfish neuromuscular junction. J. Neurosci. 14, 3688–3703. 8207482 10.1523/JNEUROSCI.14-06-03688.1994 PMC6576941

[B84] Wong A. B. Rutherford M. A. Gabrielaitis M. Pangršič T. Göttfert F. Frank T.. (2014). Developmental refinement of hair cell synapses tightens the coupling of Ca^2+^ influx to exocytosis. EMBO J. 33, 247–264. 10.1002/embj.201387110 24442635 PMC3989618

[B85] Young S. M. Neher E. (2009). Synaptotagmin has an essential function in synaptic vesicle positioning for synchronous release in addition to its role as a calcium sensor. Neuron 63, 482–496. 10.1016/j.neuron.2009.07.028 19709630

[B86] Younger M. A. Müller M. Tong A. Pym E. C. Davis G. W. (2013). A presynaptic ENaC channel drives homeostatic plasticity. Neuron 79, 1183–1196. 10.1016/j.neuron.2013.06.048 23973209 PMC3784986

[B87] Zhai R. G. Bellen H. J. (2004). The architecture of the active zone in the presynaptic nerve terminal. Physiology (Bethesda) 19, 262–270. 10.1152/physiol.00014.2004 15381754

[B88] Zhai R. G. Vardinon-Friedman H. Cases-Langhoff C. Becker B. Gundelfinger E. D. Ziv N. E.. (2001). Assembling the presynaptic active zone: a characterization of an active one precursor vesicle. Neuron 29, 131–143. 10.1016/S0896-6273(01)00185-4 11182086

